# TNF-α Involvement in Insulin Resistance Induced by Experimental Scorpion Envenomation

**DOI:** 10.1371/journal.pntd.0001740

**Published:** 2012-07-17

**Authors:** Aouatef Ait-Lounis, Fatima Laraba-Djebari

**Affiliations:** 1 Laboratory of Cellular and Molecular Biology, Faculty of Biological Sciences, University of Sciences and Technology Houari Boumediene (USTHB), Algiers, Algeria; 2 Laboratory of Research and Development on Venoms, Pasteur Institute of Algeria, Algiers, Algeria; Venezuela

## Abstract

**Background:**

Scorpion venom induces systemic inflammation characterized by an increase in cytokine release and chemokine production. There have been few experimental studies assessing the effects of scorpion venom on adipose tissue function *in vivo*.

**Methodology/Principal Findings:**

To study the adipose tissue inflammation (ATI) induced by *Androctonus australis hector* (*Aah*) venom and to assess possible mechanisms of ATI, mice (n = 6, aged 1 month) were injected with *Aah* (0.45 mg/kg), toxic fraction of *Aah* (FTox-G50; 0.2 mg/kg) or saline solution (control). Inflammatory responses were evaluated by ELISA and cell sorting analyses in adipose tissue 45 minutes and 24 hours after injection. Quantitative real-time PCR was used to assess the regulation of genes implicated in glucose uptake. The titers of selected inflammatory cytokines (IL-1β, IL-6 and TNF-α) were also determined in sera and in insulin target tissues. The serum concentration of IL-1β rose 45 minutes after envenomation and returned to basal level after 24 hours. The pathophysiological effects of the venom after 24 hours mainly involved M1-proinflammatory macrophage infiltration in adipose tissue combined with high titers of IL-1β, IL-6 and TNF-α. Indeed, TNF-α was strongly induced in both adipose tissue and skeletal muscle. We studied the effects of *Aah* venom on genes implicated in insulin-stimulated glucose uptake. Insulin induced a significant increase in the expression of the mRNAs for hexokinase 2 and phosphatidylinositol 3-kinase in both skeletal muscle and adipose tissue in control mice; this upregulation was completely abolished after 24 hours in mice envenomed with *Aah* or FTox-G50.

**Conclusions/Significance:**

Our findings suggest that *Aah* venom induces insulin resistance by mechanisms involving TNF-α-dependent Map4k4 kinase activation in the adipose tissue.

## Author Summary

*Androctonus australis hector* (*Aah*) is the scorpion most frequently causing serious human envenomation. In Algeria, *Aah* is responsible for approximately 50,000 cases of scorpion envenomation per year. The *Aah* sting causes multi-system failure that may be fatal; the manifestations include cardiopulmonary abnormalities, lung edema and inflammation. In addition, hyperglycemia and hyperinsulinemia have been described in scorpion-envenomed animals. The mechanisms causing systemic and local inflammation are poorly understood. Here, we report that *Aah* venom causes pronounced upregulation of *TNF-α, IL1-β* and *IL-6* expression in the adipose tissue, exacerbating inflammation. As the inflammatory state intensifies, 24 hours after envenomation, *TNF-α* and other factors are upregulated, and *Map4k4* expression increases, blunting the insulin response in adipocytes by decreasing Hexokinase 2 expression. Administration of TNF-α inhibitor following the envenomation reduces Map4k4 expression and restores glucose uptake in adipose tissue. These findings provide coherent evidence linking *Aah* venom-induced adipose tissue inflammation to insulin resistance. The value of TNF-α inhibitors as a treatment complementary to anti-scorpion venom immunotherapy should be evaluated clinically.

## Introduction

Scorpion venoms induce systemic inflammation associated with an increase in cytokine release and chemokine production [1]–[3]. *Androctonus australis hector (Aah)* venom induces high plasma concentrations of proinflammatory cytokines including interleukin 1 beta (IL-1β), interleukin 6 (IL-6) and tumor necrosis factor alpha (TNF-α) [4], and sympathetic tone is activated by experimental envenomation [5]. Several studies report that the sympathetic nervous system regulates the expression of several adipo-cytokines through adipocyte beta-adrenergic receptor [6], [7].

Adipose tissue secretes various cytokines including TNF-α, IL-6 and adipokines such as leptin and adiponectin involved in glucose metabolism and insulin resistance [8]. Overproduction of TNF-α in both adipose tissue and skeletal muscle contributes to insulin resistance [9]. Furthermore, TNF-α can stimulate the production of other cytokines and chemokines, such as IL-6 and Monocyte Chemoattractant Protein 1 (MCP1), which can induce insulin resistance [10], [11]. TNF-α selectively stimulates the expression of a key component of its own signaling pathway, Mitogen-activated protein 4 kinase isoform 4 (Map4k4), through a TNFR1-dependent mechanism to induce insulin resistance in adipose tissue [12].

Hyperglycemia and hyperinsulinemia have been reported in scorpion envenomed animals [13]. Although the biological activity of scorpion venom on insulin resistance is clearly established, the mechanisms involved are unknown. We have investigated the effects of scorpion venom on glucose uptake in adipose tissue. We tested the contribution, if any, of TNF-α to the modulation of insulin sensitivity after envenomation. We found that following venom injection, TNF-α increases Map4k4 expression in adipose tissue, promoting insulin resistance. The use of a chemical inhibitor (etanercept) of TNF-α binding to its receptor reduced Map4k4 expression and restored the glucose uptake in adipose tissue following envenomation.

## Materials and Methods

### Venom and animal experiment

#### Ethics statements

All experiments involving animals were carried out according to the European Community rules of the Ethical Committee for Animal Welfare. The study was approved by the Algerian National Agency of Research and Development in Health (ANDRS) which supports our project. AAL is authorized to perform experiments on vertebrate animals (authorization delivered by the Veterinary school of Algiers and by the Swiss Federal and Cantonal veterinary authorities).

#### Venoms

Lyophilized crude *Aah* venom was prepared as described [14] in the Research and Development Laboratory on Venoms of the Pasteur Institute of Algeria. Venom was collected from animals, all trapped in the same area of the country, lyophilized and stored at 4°C. The toxic fraction of *Aah* venom (FTox-G50) was isolated from the *Aah* venom by gel filtration through Sephadex G50; its homogeneity was tested by SDS-PAGE and its lethal potency was determined as described by Laraba-Djebari and Hammoudi [14].

#### Animal experiment

NMRI mice were divided into three groups (6–10 mice per group), and subcutaneously injected with: a sublethal dose of *Aah* venom (0.45 mg/kg body weight), FTox-G50 (0.2 mg/kg body weight), or 200 µl of physiological saline solution (0.9% NaCl). Some mice (n = 6) were injected by the i.p. route with a TNF-α antagonist (etanercept; 1 mg/kg body weight; Wyeth Pharmaceuticals SA, Zoug, Switzerland), 1 hour before envenomation. Animals were killed 45 min or 24 hours after injection of the toxic samples and adipose tissue and quadriceps skeletal muscle were collected.

### Intraperitoneal glucose and insulin tolerance tests

Mice were injected with *Aah* venom or the toxic fraction (FTox-G50), fasted for 6 hours and injected intraperitoneally with 25% glucose (1.5 g/kg) for glucose tolerance test (GTT) or with insulin (0.75 units/kg, Actrapid, Novo Nordisk, Denmark) for insulin tolerance test (ITT). Tail-vein blood was sampled at baseline and various times thereafter and the glucose concentration determined with a blood glucose meter (Accu-Check, Roche, Dublin, Ireland). Blood glucose was similarly determined in control and injected mice in non-fasting conditions. Insulin concentrations in serum samples were assayed by mouse ELISA (Linco Research, Inc., St Charles, MO) according to the manufacturer's instructions.

### Adipose tissue histology and morphometry

Tissue extracts from animals injected with *Aah* venom or FTox-G50 were immersed in 4% formol for 24 hours at room temperature and processed according to standard procedures for hematoxylin/eosin (HE) staining. Paraffin-embedded sections (5 µm thick) were prepared, washed in xylene and dehydrated with a series of ethanol washes. Adipose tissue sections spaced 200 µm apart were processed with HE staining. The sections were visualized with a bright field microscope and the pictures analyzed with Motic Software (Motic Images 2000, Version 3.2.0). Photographs were obtained using a ×40 objective for measuring adipocyte cell area and were analyzed using Image J software. Measurements were made on 15–25 adipocytes per section and on 3–4 sections covering the entire adipose tissue of each mouse.

### Isolation of stromal vascular cell (SVC) and macrophage sorting

Epididymal adipose tissues were rinsed three times in Phosphate-Buffered Saline (PBS), and then minced in fresh Hank's Balanced Salt Solution (HBSS) containing 1 mg/ml collagenase D and 2 ng/ml DNase I (Roche, France), incubated for 15 min at 37°C and subjected to vigorous pipetting. The resulting cell suspensions were filtered through a 70 µm-pore size mesh and centrifuged at 500 g for 5 min. The pellet (SVC) preparation, was then incubated with erythrocyte lysis buffer for 5 min, centrifuged (600 g; 5 min) and resuspended in FACS buffer. The SVCs were incubated with Fc block for 15 min at 4°C and stained with fluorescently labeled primary antibodies for 15 min at 4°C. F4/80-biotinylated FACS antibodies were purchased from AbD Serotec (Raleigh, NC); CD11b-PE and CD11c-FITC FACS antibodies were from BD Biosciences. The cells were gently washed twice and resuspended in FACS buffer, then analyzed using a FACS Aria flow cytometer (BD Biosciences, Switzerland).

### Quantitative RT-PCR

Total RNA was extracted from adipose tissue using TRIzol (Invitrogen, Carlsbad, CA). cDNA was synthesized from 1 µg RNA using random hexamer primers and Superscript II (Invitrogen, Carlsbad, CA). Quantification by PCR was performed using the iCycler iQ Real-Time PCR Detection System (Bio-Rad, Philadelphia, USA) and iQ SYBR green Supermix (Bio-Rad, Philadelphia, USA). Results were quantified by comparison to a standard curve generated with serial dilutions of a reference cDNA preparation and were normalized with respect to TATA-binding protein (TBP) mRNA. All PCR experiments were repeated at least three times. The primers used are listed in Table 1. Accession numbers for all sequences used are listed in Table S1.

**Table 1:** Primers used for real-time RT-PCR.

mRNA	*orientation	sequence
*Hk2*	F	5′-GTGAGCCATCGTGGTTAAGC-3′
	R	5′-GCGAGGCGATCATCTTGTTG-3′
*Map4k4*	F	5′-CATCTCCAGGGAAATCCTCAGG-3′
	R	5′-TTCTGTAGTCGTAAGTGGCGTCTG-3′
*IL-1β*	F	5′-GCTGAAAGCTCTCCACCTCA-3′
	R	5′-CCCAAGGCCACAGGTATTTT-3′
*IL-6*	F	5′-GAGGATACCACTCCCAACAGACC-3′
	R	5′-AAGTGCATCATCGTTGTTCATACA-3′
*IL-10*	F	5′-GAATTCCCTGGGTGAGAAGC-3′
	R	5′-CTCTTCACCTGCTCCACTGC-3′
*Pik3r2*	F	5′-GAGAGCCCTGTCTTCAGTC-3′
	R	5′-CACTGCCGTCCGAGTTTAC-3′
*TNF-α*	F	5′-CCACGCTCTTCTGTCTACTGAACT-3′
	R	5′-GGGTCTGGGCCATAGAACTG-3′
*Tbp*	F	5′-ATGCTGAATATAATCCCAAGCGA-3′
	R	5′-GAAAATCAACGCAGTTGTCCG-3′

### Insulin-stimulated glucose uptake into adipose tissue explants

Adipose tissue from controls and *Aah* venom- or FTox-G50- envenomed mice were placed in 24-well plates (100 mg of tissue/well) with 1 mL of PBS+0.2% Bovine Serum Albumin (BSA), and then stimulated with insulin (100 nmol/L) for 1 hour. The tissues were then treated with TRIzol (Invitrogen, Carlsbad, CA) to lyse cells for total RNA extraction. Quantitative RT-PCR was used to measure the mRNAs for selected genes implicated in insulin-stimulated glucose uptake.

### Measurement of cytokine concentrations

Serum samples were collected from the retro-orbital sinus under anesthesia by injection of sodium pentobarbital. Adipose tissue extracts (0.5 g of tissue) were homogenized with a polytron homogenizer in 1 ml buffer containing PBS and 0.04% Tween 80. These tissue samples were centrifuged at 10,000 *g* for 10 min and the supernatants collected. TNF-α, IL-1β and IL-6 concentrations in the tissue supernatants or in the serum samples were determined with ELISA kits (eBioscience, San Diego, CA) according to the manufacturer's instructions.

### Statistical analysis

Data are reported as means ± s.e.m. Two-way ANOVA tests or unpaired t tests and GraphPad Prism 5 (GraphPad Software, San Diego, CA) were used for comparisons between groups. Area under the curve analysis was performed on GTT and ITT curves using Graphpad Prism 5 Software. Statistical significance is indicated * for P<0.05, **P<0.01, and ***P<0.001.

## Results

### Impaired glucose tolerance and decreased action of insulin on the peripheral tissues of *Aah* venom- and FTox-G50- injected mice

**Figure 1:**
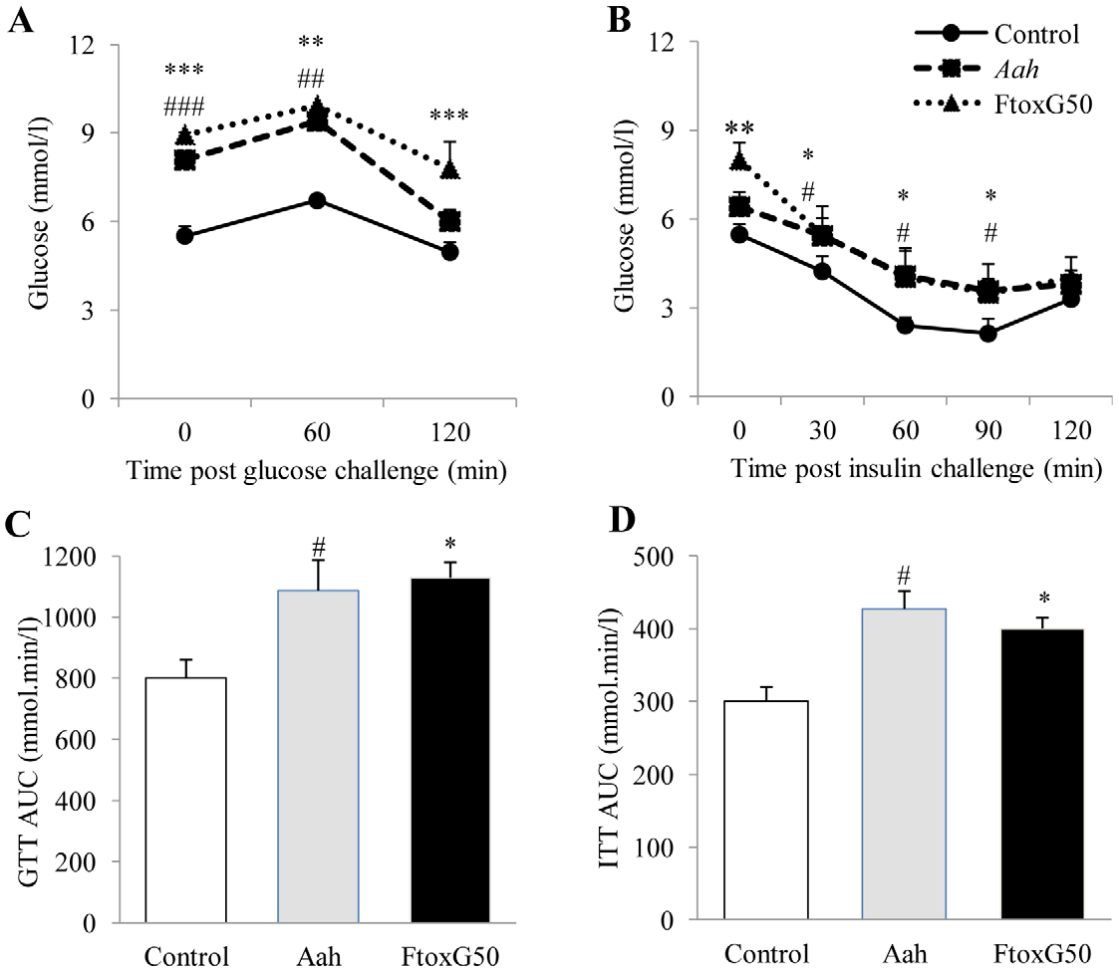
Glucose tolerance test (GTT) and insulin tolerance test (ITT) after *Aah* venom and FTox-G50 injection. (A): GTT (1.5 g/kg glucose) was performed in 6-h fasted control mice and mice injected with *Aah* venom and FTox-G50 fraction. (B): ITT (0.75 units/kg insulin) was performed in 6-h fasted control, *Aah* and *FTox-G50* animals (•, control; ▪, *Aah*; ▴, *FTox-G50*; *P<0.05, ***P<0.001 control versus FTox-G50; #P<0.05, ##P<0.01, n = 6). (C) and (D): Area under the curve (AUC) for control and *Aah* venom- and FTox-G50-injected animals over the course of the GTT and ITT was calculated and is expressed in mmol.min/l. Data were analyzed by two-way ANOVA.

We tested whether total *Aah* venom and its toxic fraction (FTox-G50) caused glucose intolerance. Young adult mice (1 month-old) were injected with *Aah* venom or FTox-G50, and fasted for 6 hours during the day. Fasting blood glucose levels were significantly higher in *Aah* venom- and FTox-G50-treated mice than controls (Figures 1A, 1C). Following glucose injection, blood glucose levels were twice as high in envenomated mice as in control mice. The glucose level in mice injected with *Aah* venom returned to the basal level after 120 min whereas blood glucose levels remained elevated in mice injected with FTox-G50 (Figures 1A, 1C; P<0.001). Glucose intolerance can result from the absence of glucose-stimulated insulin secretion or a decreased action of insulin in the peripheral tissues, or both. We therefore assayed plasma insulin in mice injected with *Aah* venom and FTox-G50 after 45 min and 24 hours (Table 2). Forty-five min after envenomation, the plasma insulin concentration was significantly higher in mice injected with *Aah v*enom or FTox-G50 (0.6 to 1.66 µg/l and 0.61 to 2.41 µg/l, respectively), than in controls (0.28 to 0.48 µg/l). This hyperinsulinemia persisted for 24 hours after envenomation (Table 2). Thus, *Aah* venom and FTox-G50 block the action of insulin, resulting in glucose intolerance.

**Table 2:**
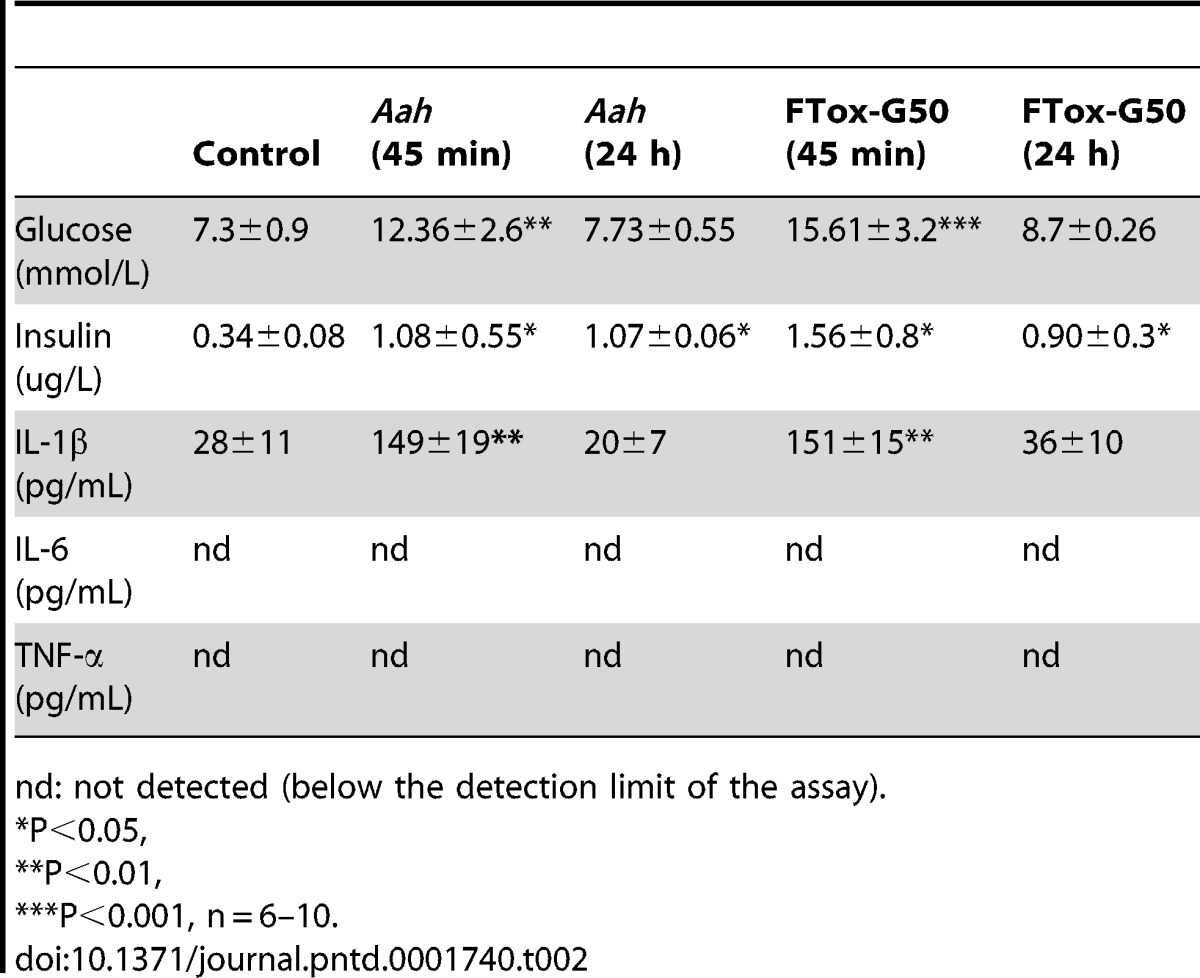
Plasma metabolic profile.

nd: not detected (below the detection limit of the assay).

*: P<0.05,

**: P<0.01,

***: P<0.001, n = 6–10.

The ability of peripheral tissues to take up glucose in response to exogenous insulin was tested. Glucose uptake in mice treated with *Aah* venom or FTox-G50 was significantly higher (P<0.05) to that in control mice (Figures 1B, 1D). These results indicate that mice injected with *Aah* venom or FTox-G50 were not deficient in glucose-stimulated insulin secretion supporting the hypothesis of insulin resistance. Plasma IL-1β concentrations 45 min post-envenomation were higher in both *Aah* venom- and FTox-G50–injected mice than controls (P<0.05), but returned to basal values after 24 hours (Table 2). This finding agrees well with the higher blood glucose concentration in both *Aah* venom- and FTox-G50 injected mice than control mice (Table 2).

### Strong adipose tissue inflammation in *Aah* venom and FTox-G50 injected mice

**Figure 2:**
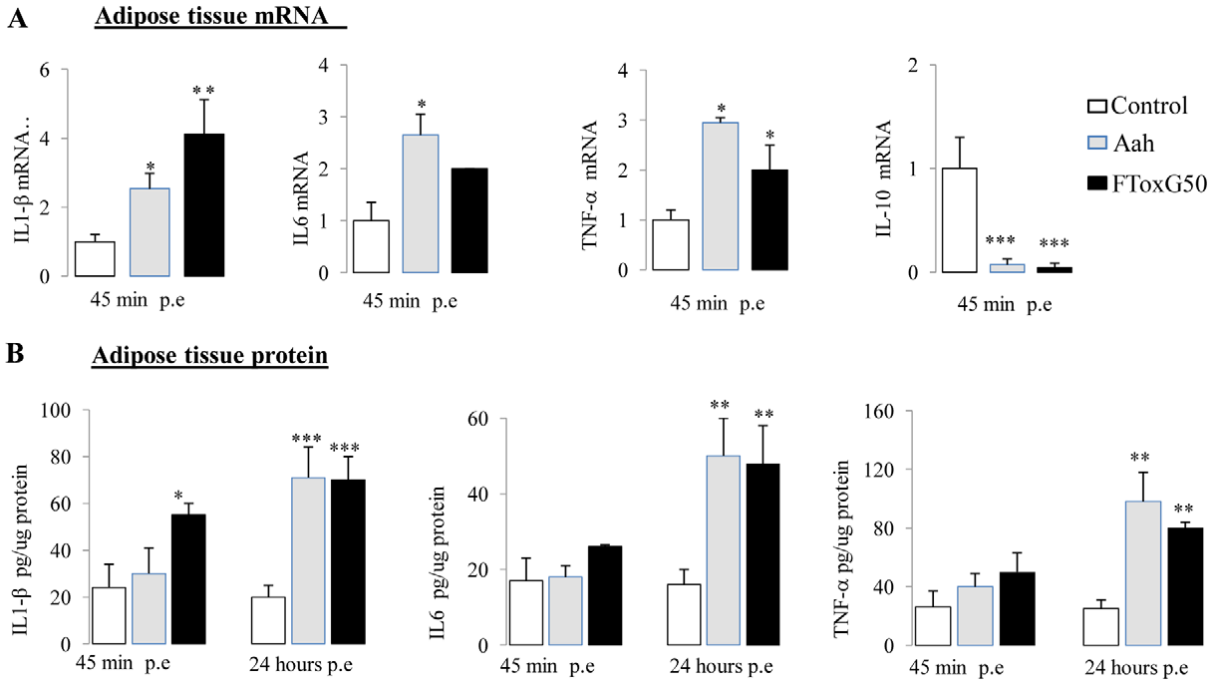
Adipose tissue inflammatory cytokine expression in control, *Aah* venom- and FTox-G50-injected mice. (A) *IL-1β, IL-6, TNF-α* and *IL-10* mRNA expression was measured by qRT-PCR in total adipose tissue RNA at 45 minutes after *Aah* and FTox-G50 envenomation. The results were normalized using *Tbp* mRNA and were expressed relative to the levels found in control mice. (B) Levels of proinflammatory IL-1β, IL-6, and TNF-α secretion were measured by ELISA in adipose tissue at 45 minutes and 24 hours post-envenomation. Data were analyzed by two-way ANOVA;*P<0.05, **P<0.01,***P<0.001, n = 6–10.

Adipose tissue inflammation was analyzed in control mice and mice injected with *Aah* venom or FTox-G50 after 45 min and 24 hours. Significant mRNA upregulation of several proinflammatory cytokines (*IL-1β, IL-6* and *TNF-α*) was observed (Figure 2A). Similar to previous study [4], mRNA down-regulation of *IL-10* was observed rapidly after envenomation (Figure 2A). To evaluate whether increased adipose cytokine mRNA expression resulted in increased protein production, we have measured IL-1β, IL-6 and TNF-α cytokine release from *Aah* venom and FTox-G50 adipose tissue *ex vivo* compared with control tissue (Figure 2B). IL-1β, IL-6 and TNF-α concentrations in adipose tissue 24 hours after envenomation were significantly higher than those in controls (P<0.001 for IL-1β; P<0.01 for IL-6 and TNF-α), although the circulating IL-1β concentration returned to its basal value (Figure 2 B, Table 2). These findings demonstrate that there is a local inflammatory profile in adipose tissue 24 hours following injection of *Aah* venom and FTox-G50.

### *Aah* venom and FTox-G50 injection increase the TNF-α concentration in skeletal muscle

**Figure 3:**
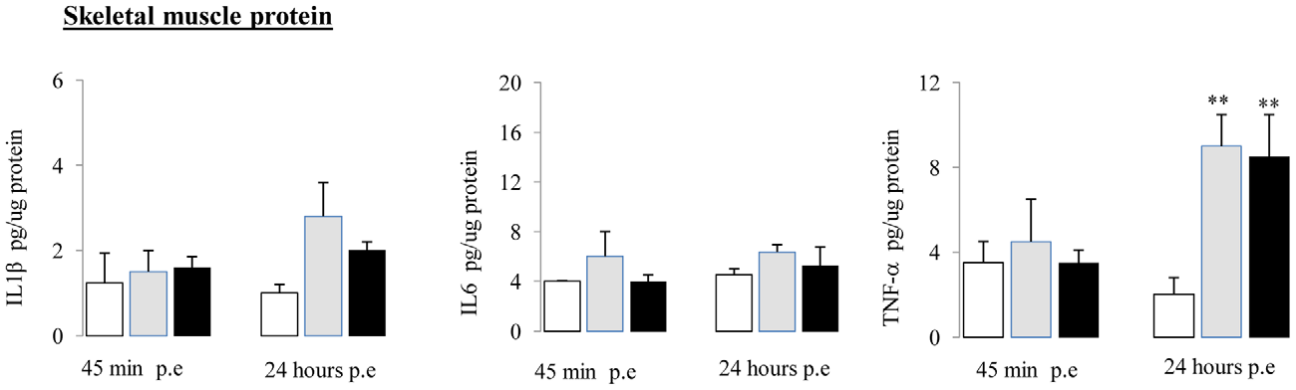
Inflammatory cytokine expression in skeletal muscle from control, *Aah* venom- and FTox-G50-injected mice. IL-1β, IL-6, and TNF-α in skeletal muscle were assayed by ELISA, 45 min and 24 hours post-envenomation. **P<0.01, n = 6.

Quadriceps skeletal muscle inflammation was analyzed in explants from control, *Aah* venom- and FTox-G50-injected mice 45 min and 24 hours after envenomation. The TNF-α concentration was higher in animals injected with venom or its toxic fraction 24 hours post-envenomation than in controls (Figure 3). No significant changes were observed for the production of either IL-1β or IL-6 in mice following *Aah* or FTox-G50 injection (Figure 3).

### Analysis of adipose tissue morphology and pro-inflammatory macrophage infiltration in adipose tissue after envenomation

**Figure 4:**
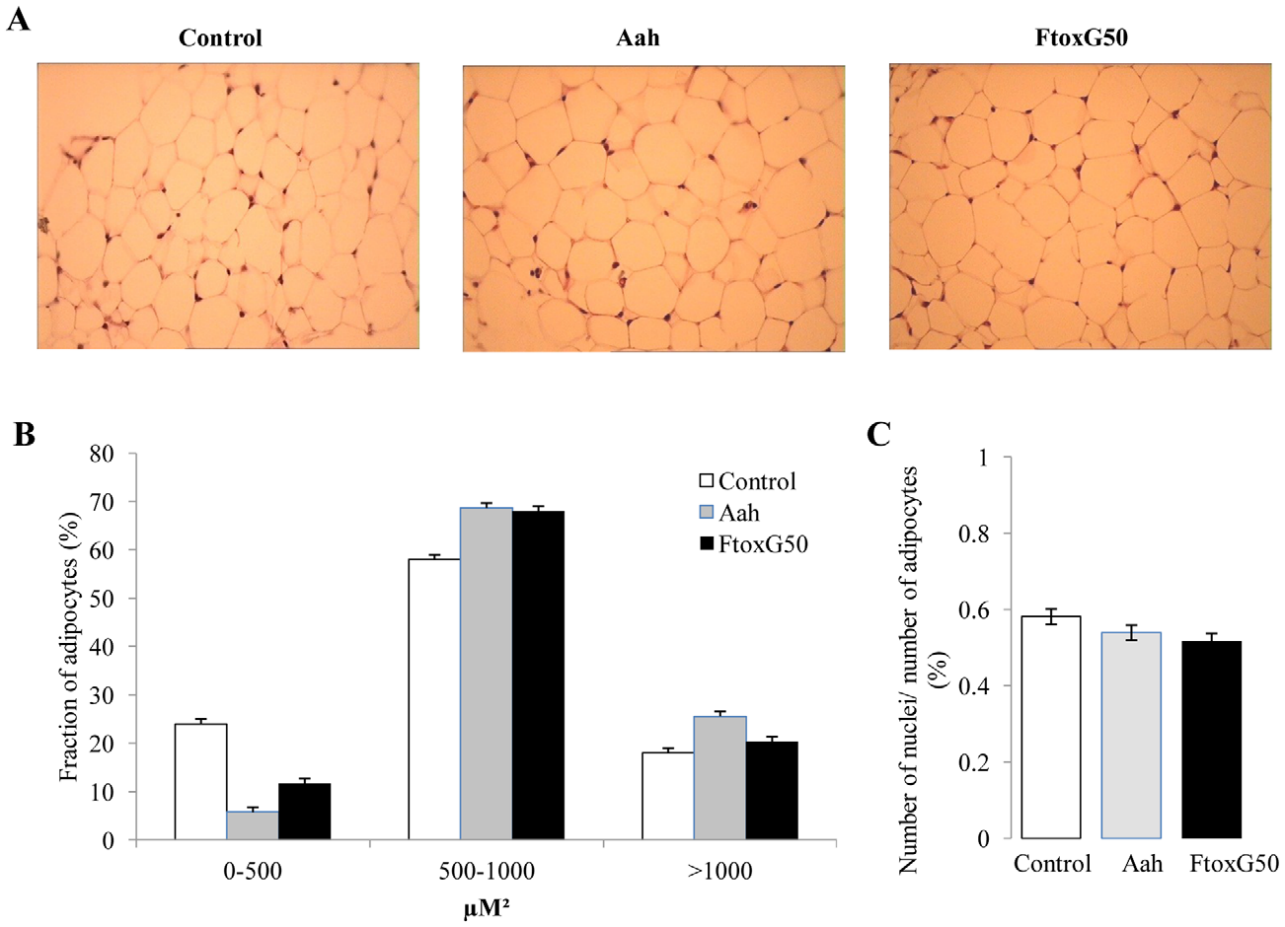
*Aah* venom and FTox-G50 effect on adipose tissue formation. (A). Adipocytes in white adipose tissue were visualized by hematoxylin/eosin staining. Representative HE staining in control and 24 hours after *Aah* and FTox-G50 injection are shown. (B) The volume densities of adipocytes were quantified in control samples and in samples prepared 24 hours after envenomation. (C) The number of nuclei per adipocyte was counted.

Adipose tissue morphology was analyzed 24 hours following *Aah* venom and FTox-G50 injection. HE staining of adipose tissue sections revealed no difference in adipocyte size or morphology between treated and control animals (Figures 4A, 4B); total number of nuclei was unaffected in the injected mice (Figure 4C).

**Figure 5:**
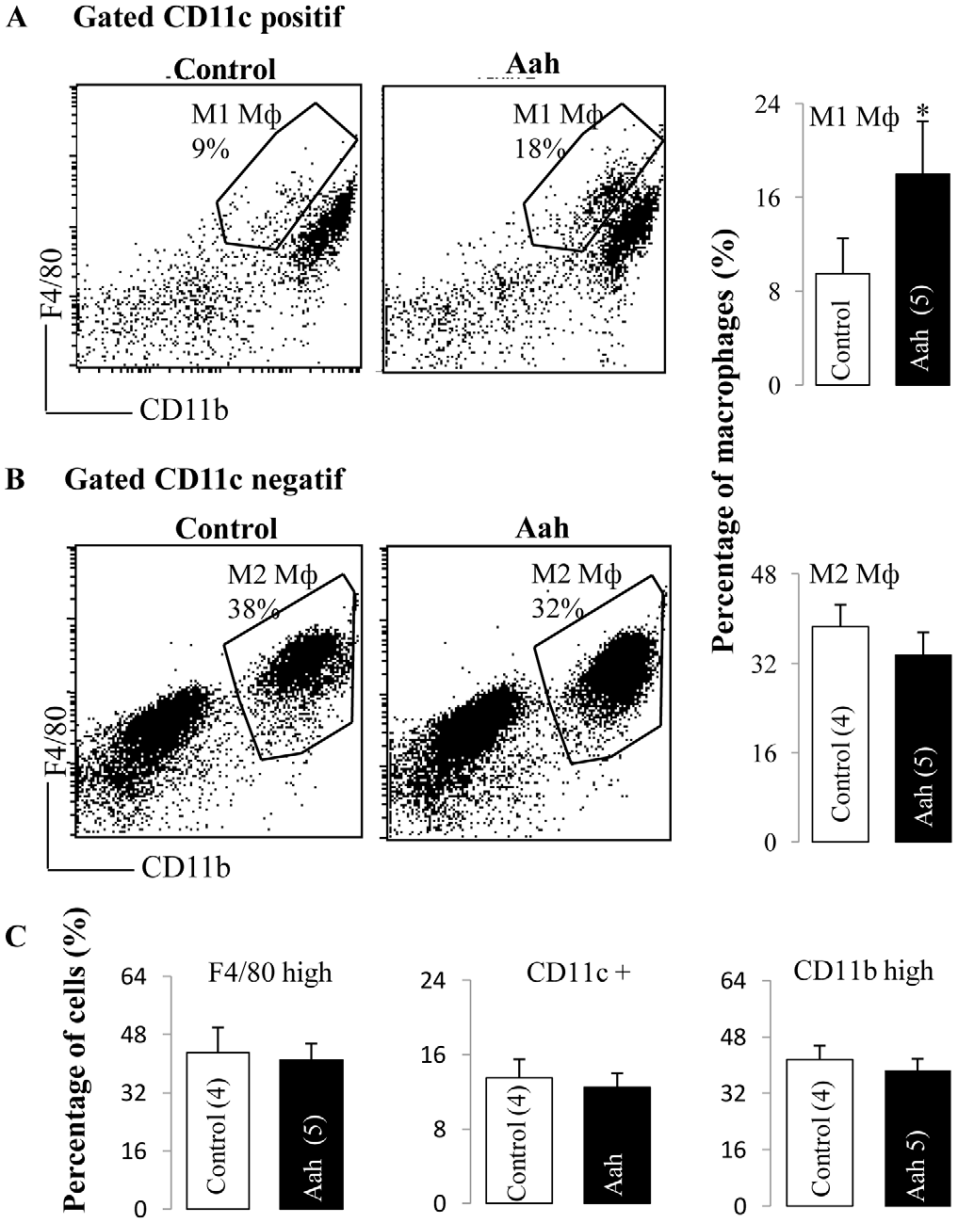
Recruitment of macrophages into adipose tissue and immunogenic phenotype of macrophages isolated from adipose tissue. Adipose tissue was harvested from control and *Aah*-injected mice after 24 hours and stromal vascular cells (SVCs) were separated from tissue by collagenase digestion. SVCs were stained for F4/80, CD11b, and CD11c cell surface proteins and analyzed by flow cytometry. (A) Representative flow-cytometry profiles for the expression of M1 Macrophages (F4/80 high CD11b high CD11b+) from control and *Aah*-injected mice. Numbers indicate the percentages of M1 cells found in the indicated gates. Histograms show the quantifications of the percentages of M1 macrophages. (B) Representative flow-cytometry profiles for M2 macrophages (F4/80 high CD11c high CD11c negatif) from control and *Aah*-injected mice. Numbers indicate the percentage of cells found in the indicated gates. (C) Histograms show the quantifications of the percentage of F4/80 high alone, CD11b high alone, and CD11c positive cells. The means, standard deviations, and numbers of mice are indicated. Analyses were by two-way ANOVA,*, p<0.05.

Adipose tissue inflammation is associated with an increased number of adipose tissue macrophage (ATMs), referred to as M1-proinflammatory macrophages in which ATMs express high levels of CD11c, F4/80 and CD11b [15]. Flow cytometry analysis revealed that the total number of triple positive (F4/80 high, CD11b high and CD11c+) cells in adipose tissue was high 24 hours after envenomation, indicating that M1 proinflammatory macrophages accumulated in adipose tissue rapidly after envenomation (Figure 5 A). The number of CD11c-negative ATMs (F4/80 high, CD11b high, CD11c−), referred to as M2-non-inflammatory macrophages, after *Aah* envenomation was lower than that in controls (Figure 5B). These results indicated that proinflammatory M1 ATMs infiltrate adipose tissue in mice injected with *Aah* venom.

### The injection of FTox-G50 induces insulin resistance in adipose tissue

**Figure 6:**
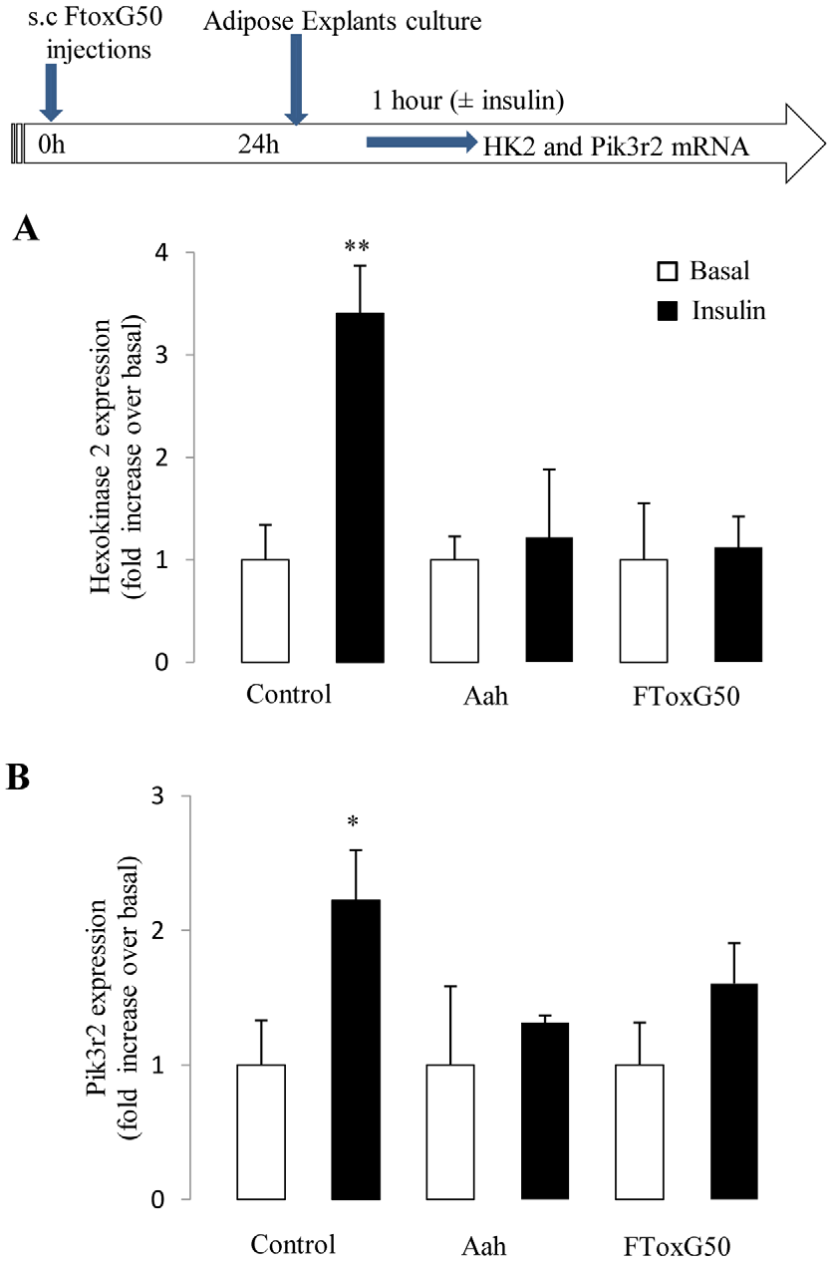
*Ex vivo* assessment of adipose tissue insulin sensitivity in FTox-G50-injected mice. Adipose explants from controls and mice treated 24 hours earlier with *Aah* venom or FTox-G50 were harvested and stimulated with or without insulin (100 nmol/L) *ex vivo* for 1 hour, as shown in the scheme (top). The control graph represents the ability of insulin to promote glucose uptake into adipose tissue. Insulin-induced changes in *Hk2*. (A) and *Pik3r2* expression (B) mRNA levels were determined by quantitative RT-PCR in adipose explants from controls and mice injected with native and toxic fraction of venom. Ratio of Hk2 and Pik3r2 mRNA expression in response to insulin over basal level (non-insulin stimulated) in adipose tissue for each individual mouse is represented. Data were analyzed by unpaired t test; *P<0.05, **P<0.01..n = 6.

We investigated whether the inflammation of adipose tissue resulting from FTox-G50 injection contributed to adipose-specific insulin sensitivity. We studied the expression of two genes involved in glucose metabolism, Hexokinase 2 (*Hk2*) and Phosphatidylinositol 3-kinase, regulatory subunit, polypeptide 2 (*Pik3r2*). Insulin stimulation resulted in the increased expression of *Hk2* mRNA in the adipose tissue of control mice (Figure 6A); this correlated with an increased insulin-induced *Pik3r2* expression (Figure 6B). In contrast, *Aah* venom or FTox-G50 administration completely blocked the induction by insulin of both *Hk2* and *Pik3r2* mRNA expression (Figures 6A and 6B). Therefore, the venom causes insulin resistance on glucose metabolism.

### TNF-α inhibition prevents insulin resistance in FTox-G50-treated adipose tissue

**Figure 7:**
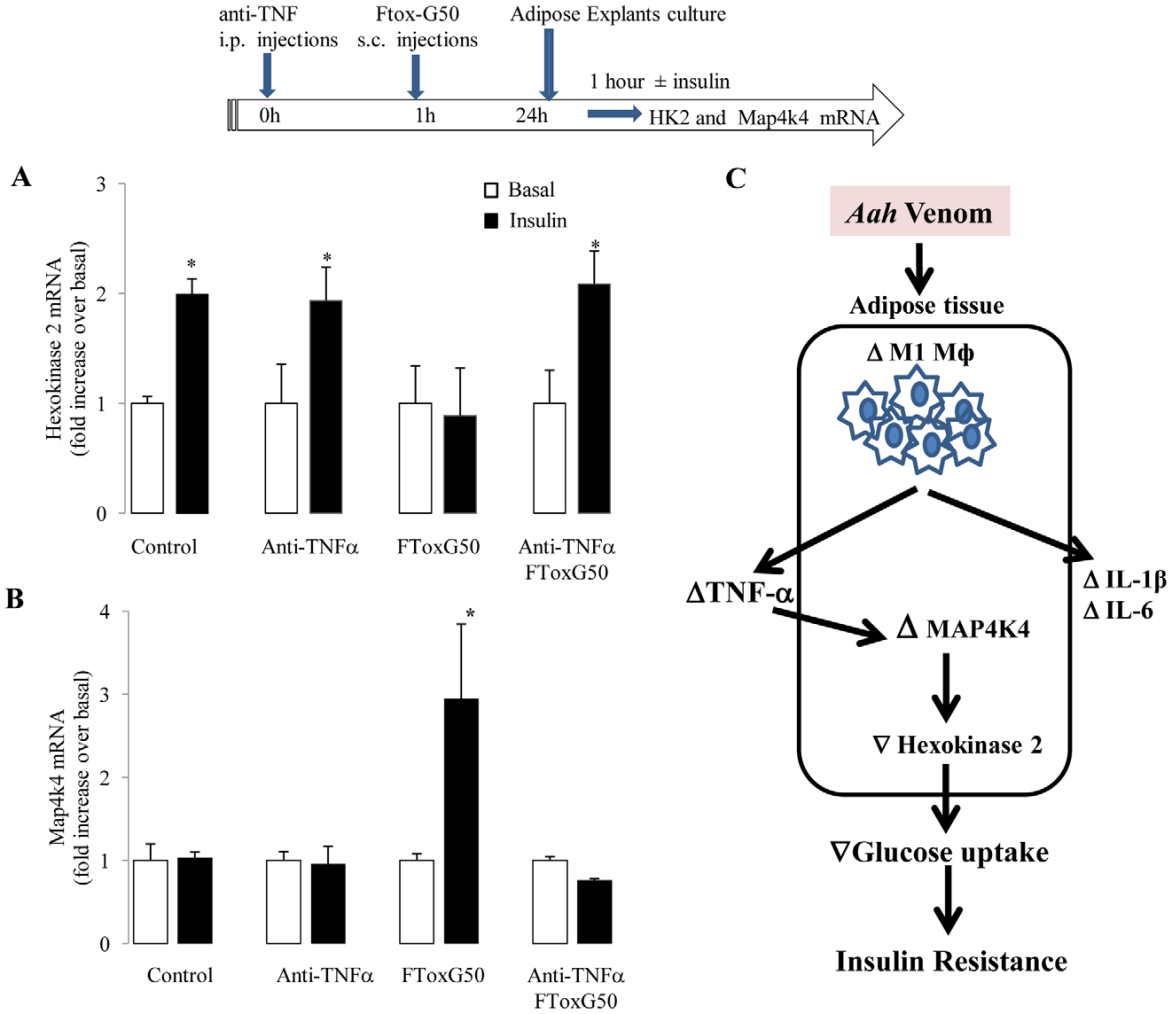
Anti-TNF-α treatment effect on insulin sensitivity in adipose tissue from *FTox-G50*-treated mice. Four-week-old mice were injected with etanercept (anti-TNFα, 1 mg/kg/injection) one hour before injection of FTox-G50. Twenty-four hours later, adipose explants were harvested and stimulated with insulin (100 nmol/L) *ex vivo* for 1 hour as shown in the scheme (top). (A) Insulin-induced changes in Hk 2 mRNA levels in adipose explants were determined by quantitative RT-PCR. (B) Insulin-induced changes in Map4k4 mRNA levels in adipose explants were determined by quantitative RT-PCR. Ratio of Hk2 and Map4k4 mRNA expression in response to insulin over basal level (non-insulin stimulated) in adipose tissue for each individual mouse is represented. Data were analyzed by unpaired t test*, P<0.05. n = 6. (C) Cellular mechanisms of decreased glucose uptake after *Aah* injection. *Aah* venom causes pronounced upregulation of TNF-α, IL1-β and IL-6 expression in the adipose tissue, exacerbating the inflammatory state. As the inflammatory state intensifies 24 hours after envenomation, TNF-α and other factors are upregulated causing activation of Map4k4 expression and blunting the insulin response in adipocytes by decreasing of Hk2 expression. Up and down arrows indicate increased and decreased expression, respectively.

We tested whether TNF-α inhibition could prevent insulin resistance in adipose tissue. We treated 1-month-old mice with a chemical inhibitor directed against TNF-α binding (etanercept). In control adipose explants, insulin increased *Hk2* mRNA expression by 2 fold (Figure 7A; P<0.01). Anti-TNF-α treatment in control mice had no affect on insulin-induced *Hk2* mRNA expression by contrast, FTox-G50 injection completely blocked insulin-induced *Hk2* mRNA expression (Figures 6A; 7A). However, FTox-G50-induced insulin resistance on glucose metabolism was abolished by anti-TNF-α treatment, suggesting that insulin resistance induced by venom is TNF-α dependent.

We tested whether anti-TNF-α treatment of FTox-G50 mice affected Map4k4 kinase expression in adipose tissue. Map4k4 mediates the effects of TNF-α in adipose tissue and skeletal muscle [12], [16]. As expected, in control mice, basal and insulin-stimulated Map4k4 expression were unaffected by anti-TNF-α treatment (Figure 7B). Injection of FTox-G50 resulted in a 3-fold increase in Map4k4 expression 24 hours later (P<0.01), and anti-TNF-α treatment of FTox-G50-injected mice prevented this Map4k4 upregulation (Figure 7B). Our findings for the effects of the TNF-α inhibitor etanercept and for Map4k4 expression strongly suggest that the venom-induced insulin resistance is TNF-α-dependent and was mediated at least in part by Map4k4 activation in adipose tissue (Figure 7C).

### TNF-α inhibition does not prevent FTox-G50-induced insulin resistance in skeletal muscle

**Figure 8:**
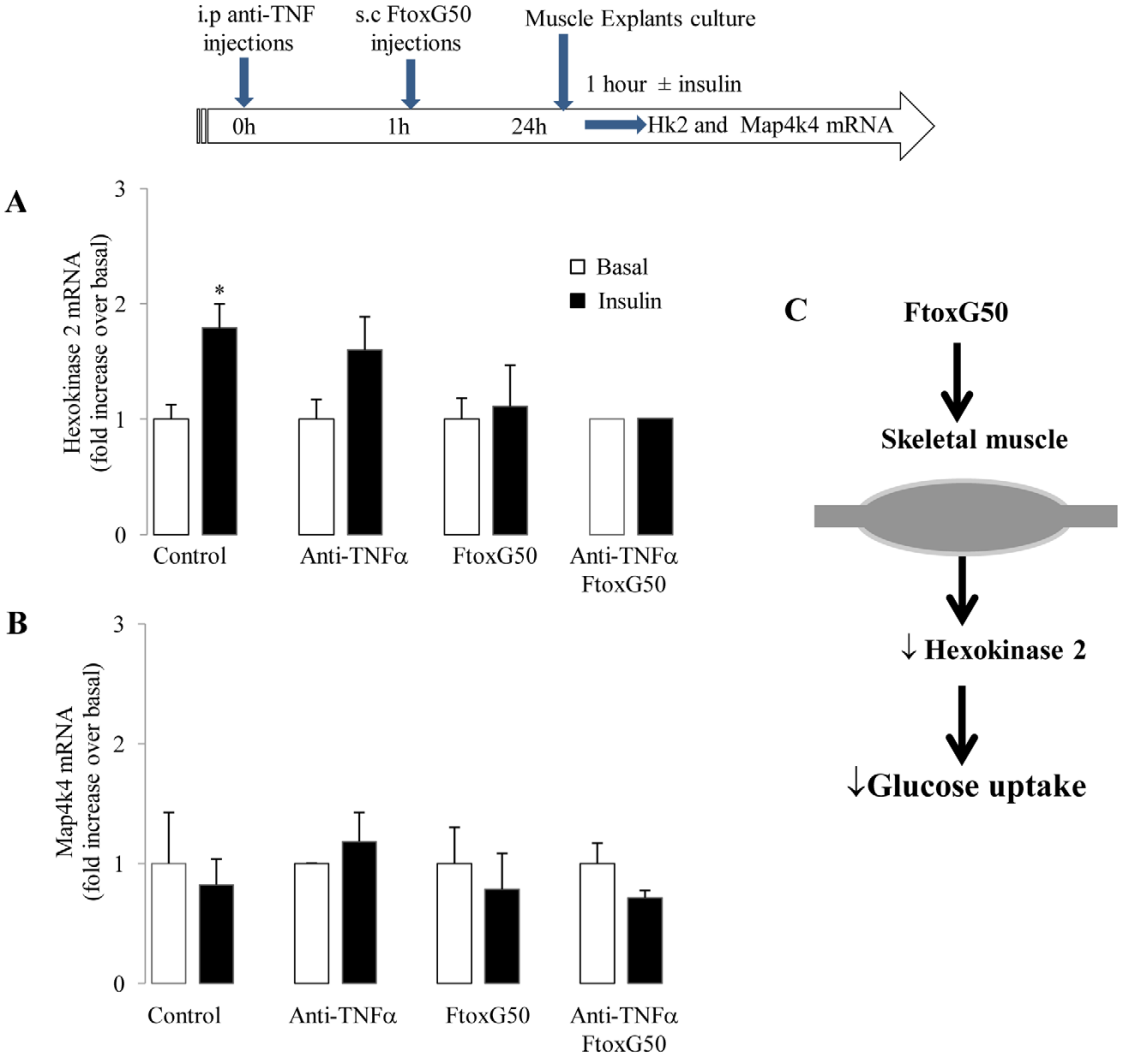
Effects of anti-TNF-α treatment on insulin sensitivity in skeletal muscle after FTox-G50 injection. Four-week-old mice were injected with etanercept (anti-TNFα, 1 mg/kg/injection) and one hour before FTox-G50-injection. Twenty-four hours later, skeletal muscle explants were harvested and stimulated with insulin (100 nmol/L) *ex vivo* for 1 hour as shown in the scheme (top). (A) Insulin-induced changes in Hk2 mRNA levels in skeletal muscle were determined by quantitative RT-PCR. (B) Insulin-induced changes in Map4k4 mRNA levels in skeletal muscle were determined by quantitative RT-PCR. Ratio of Hk2 and Map4k4 mRNA expression in response to insulin over basal levels (non-insulin stimulated) in adipose tissue and skeletal muscle for each individual mouse is represented. (C) Schematic diagram of decreased glucose uptake in skeletal muscle after FTox-G50 injection; up and down arrows indicate increased and decreased expression, respectively.

We tested whether FTox-G50 caused insulin resistance in skeletal muscle and whether anti-TNFα treatment could prevent any such effect. In skeletal muscle explants, insulin treatment increased *Hk2* mRNA expression (1.7 fold more than in controls; Figure 8A). In control mice treated with anti-TNF-α, insulin treatment slightly but not significantly increased in *Hk2* expression (Figure 8A). FTox-G50-induced insulin resistance in skeletal muscle was not abolished by anti-TNF-α treatment and *Map4k4* expression was not activated in skeletal muscle of mice injected with FTox-G50 (Figure 8B). Therefore, FTox-G50-induced insulin resistance in skeletal muscle appeared to be TNF-α-independent, and is presumably mediated by other factors (Figure 8C).

## Discussion

Scorpion venoms contain a diversity of neurotoxins, including two major polypeptide populations. One consists of several classes of long-chain peptides (60–70 amino acid residues) affecting Na^+^ channels [17], and the other includes short-chain toxins affecting K^+^ [18], Cl^−^ [19] and Ca2^+^ channels [20]. All these toxins have direct effects on the ion permeability of excitable cells. The venom of the scorpion *Buthus occitanus tunetanus* (*Bot*) also contains compounds that activate other cell functions in non-excitable cells, such as adipocytes [21], [22]. The addition of *Bot* venom to the culture media of 3T3-L1 adipocytes or freshly dissociated rat adipocytes rapidly increases lipolysis, as indicated by glycerol release, and does so in a dose-dependent manner [22].

In this work, we demonstrate that scorpion venom can reduce insulin sensitivity in mice. This further strengthens the idea that venom may cause insulin resistance, as described previously [13]. Our findings confirm present reports that scorpion venom induces systemic and local inflammation. In particular, we demonstrate that following envenomation, the expression pattern of proinflammatory cytokines (IL-1β, IL-6, TNF-α) changes substantially in adipose tissue concomitant with infiltration by pro-inflammatory macrophages. Interestingly, TNF-α treatment reduces Map4k4 expression and restores glucose uptake in adipose tissue following envenomation. These observations suggest that decreased insulin sensitivity in mice injected with venom is mainly driven by TNF-α.

Hyperglycemia and hyperinsulinemia have been described in scorpion envenomed animals [13]. We observed increased in fed glucose levels 45 min after *Aah* envenomation. This hyperglycemia did not worsen over the subsequent hours following envenomation, although the hyperinsulinemia persisted many hours after envenomation. Possibly this persistence of hyperinsulinemia, most likely as a result of increasing β-cell hyperplasia, serves to compensate and regulate the glucose level such that it returns to normal values.

The effects of inflammation and infiltration of macrophages on adipose tissue function and insulin resistance have been extensively studied [23], [24]. Macrophage recruitment into adipose tissue plays a key role in the etiology of diet-induced insulin resistance [24]. The phenotypes exhibited by tissue macrophages correspond to a M1–M2 polarization state: M1 cells are defined as activated pro-inflammatory macrophages and M2 cells comprise an anti-inflammatory macrophage population. We observed that the total number of M2 cells that are positive for F4/80 and CD11b but negative for CD11c expression in l adipose tissue did not increase following envenomation, whereas the number of M1-like macrophages (F4/80 high, CD11b high, CD11c+) increased significantly. These results are consistent with the view that these proinflammatory CD11c+ macrophages are the cause of the macrophage-linked component of inflammation/insulin resistance; indeed genetic deletion of these cells is sufficient to normalize obesity-induced inflammation, glucose tolerance and insulin resistance [25]. Therefore, it is possible that the increased numbers of M1-like macrophages in adipocyte tissue in mice injected with venom explains the elevated secretion of TNF-α, IL-6 and IL-1β and thereby contributes to the low grade inflammation and insulin resistance.

Adipocytes secrete a number of molecules, including leptin, TNF-α, IL-6, and resistin, that modulate peripheral insulin sensitivity [8], [26]–[28]. Consistent with this, we found that TNF-α concentrations in adipose tissue and skeletal muscle were increased following injection of *Aah* venom or the FTox-G50 fraction. TNF-α stimulates the expression of key components of its own signaling pathway, notably Map4k4, through a TNFR1-dependent mechanism to induce insulin resistance in adipose tissue [12]. Another study has shown that insulin resistance can be abolished by Map4k4 silencing in skeletal muscle [16]. Here, we show that venom injection significantly increased Map4k4 gene expression and that inhibition of TNF-α significantly reduced *Map4k4* gene expression in adipose tissue. The specificity of TNF-α action on Map4k4 is due to the unique phosphorylation of JNK1/2 and p38 SAP kinase that leads to activation of the transcription factors c-JUN and ATF2, which in turn are required for the regulation of Map4k4 expression [12]. Our observations are consistent with these findings and indicate that the decreased insulin sensitivity observed following FTox-G50 injection is mediated by an increase in the TNF-α concentration in adipose tissue, which selectively stimulates the expression of Map4k4 to cause insulin resistance.

It may be an oversimplification to attribute adipose tissue inflammation to the effect of a single cytokine: it is likely that several cytokines act collectively to amplify the inflammatory response of adipose tissue. Indeed, we have demonstrated that the concentrations of TNF-α, IL-6 and IL-1β in adipose tissue were increased by *Aah* venom and FTox-G50 treatment. However, note that although TNF-α increased in skeletal muscle after 24 hours of envenomation, no significant changes in IL-1β and IL-6 levels were detected. The major adipocytokines IL-1β and TNF-α act synergistically to enhance NFkB activation and secretion of IL-6 in adipose tissue [28]. Furthermore, the ability of TNF-α to induce IL-6 secretion is blunted in IL-1RI−/− adipose tissue, suggesting that TNF-α-induced IL-6 is in part mediated by IL-1 [28]. The ability of TNF-α to induce IL-6 secretion has also been demonstrated in adipose tissue and skeletal muscle [16]. It is therefore plausible that increased IL-6 expression in adipose tissue after envenomation may be due to TNF-α production, suggesting that TNF-α and IL-1β work in concert to cause insulin resistance.

The mechanisms of activation of pro-inflammatory cytokines in adipose tissue following envenomation remain unclear. Adipose tissue in rodents is innervated by the sympathetic nervous system, which can regulate lipolysis, fat cell number, and the secretion of some adipocytokines, such as TNF-α and MCP1 [6], [7]. Furthermore, the activity of the sympathetic nervous system in mice increases following envenomation, an effect mediated by catecholamines [6], [7]. In addition, the activity of sympathetic nervous system can contribute to insulin resistance through effects of catecholamines on adipocytes [6], [7]. Nevertheless, our results do not rule out the possibility that the expression of adipocytokines is regulated through β-adrenergic receptors. The pharmacological profiles of molecules acting more selectively on β-adrenergic receptor subtypes strongly suggest that the lipolytic action of *Bot* venom mainly involves the β2/β1 subtype of adrenergic receptors [21].

In conclusion, we report that *Aah* venom and its toxic fraction induce M1-like macrophages accumulation, inflammation and insulin resistance in adipose tissue. We demonstrate an increase in TNF-α release causing upregulation of Map4k4 expression that disrupts the normal metabolic function of adipose tissue and thereby leads to insulin resistance. These findings suggest that pharmacological inhibition of TNF-α in animals injected with scorpion venom may restore metabolic function and subsequently improve insulin sensitivity. This study provides consistent evidence linking adipose tissue inflammation to the insulin resistance induced by *Aah* venom. We believe that it would be useful to assess the value of TNF-α inhibitors for the complementary treatment of scorpion envenomation.

## Supporting Information

Table S1 List of accession numbers/ID numbers for genes mentioned in the text.
(DOC)
