# Supplementary material for: TNF-α Involvement in Insulin Resistance Induced by Experimental Scorpion Envenomation
Source: PLoS Negl Trop Dis. 2012 Jul 17;6(7):e1740. doi: 10.1371/journal.pntd.0001740 (PMC3398957; doi:10.1371/journal.pntd.0001740)
Supplement: Table S1 — List of accession numbers/ID numbers for genes mentioned in the text. (DOC) [file pntd.0001740.s001.doc]

# Supplementary Table S1: List of Accession Numbers

| **Gene name (Gene symbol)** | **NCBI Reference** |
| --- | --- |
| Mus musculus mitogen-activated protein kinase kinase kinase kinase 4 (Map4k4) | NM_001252200.1 |
| Mus musculus hexokinase 2 (Hk2), | NM_013820.3 |
| Mus musculus interleukin 1 beta (Il1b) | NM_008361.3 |
| Mus musculus interleukin 6 | BC138766.1 (GenBank) |
| Mus musculus interleukin 10 (Il10) | NM_010548.2 |
| Mus musculus phosphatidylinositol 3-kinase, regulatory subunit, polypeptide 2 (p85 beta) (Pik3r2) | NM_008841.2 |
| Mus musculus tumor necrosis factor (Tnf) | NM_013693.2 |
| Mus musculus TATA box binding protein (Tbp) | NM_013684.3 |
